# Polymorphisms in the Estrogen Receptor Beta Gene and the Risk of Unexplained Recurrent Spontaneous Abortion

**Published:** 2017

**Authors:** Marzieh Mahdavipour, Saeed Zarei, Ramina Fatemi, Haleh Edalatkhah, Hamed Heidari-Vala, Mahmood Jeddi-Tehrani, Farah Idali

**Affiliations:** 1. Reproductive Immunology Research Center, Avicenna Research Institute, ACECR, Tehran, Iran; 2. Monoclonal Antibody Research Center, Avicenna Research Institute, ACECR, Tehran, Iran; 3. Reproductive Biotechnology Research Center, Avicenna Research Institute, ACECR, Tehran, Iran

**Keywords:** Estrogen receptor, Habitual abortion, Polymerase chain reaction, Restriction fragment length polymorphism, Single-nucleotide polymorphism

## Abstract

**Background:**

Recurrent Spontaneous Abortion (RSA) is caused by multiple genetic and non-genetic factors. Around 50% of the RSA cases have no known etiology and are considered as Unexplained RSA (URSA). Estrogens, *via* binding to their receptors, play an important role in female reproduction. This study aimed to investigate whether single nucleotide polymorphisms (SNPs; +1082G/A, +1730G/A and rs1256030 C/T) in the estrogen receptor beta (*ESR2*) gene are associated with susceptibility to URSA in a population of Iranian women.

**Methods:**

In this case-control study, the study groups consisted of 240 subjects with a history of URSA and 102 fertile women as controls. Serum levels of follicle stimulating hormone (FSH), luteinizing hormone (LH), and estradiol (E2) were measured on day 2–3 of menstrual cycle. Two functional SNPs, +1082G/A (a silent mutation in exon 5) and +1730G/A (3′ untranslated region of the exon 8), and one intron, rs1256030C/T, in the *ESR2* gene were genotyped, using polymerase chain reaction-restriction fragment length polymorphism (PCR-RFLP) analysis.

**Results:**

Serum levels of LH were significantly increased in URSA women. No significant differences in distribution of +1082G/A, +1730G/A and rs1256030C/T between URSA and control groups were observed.

**Conclusion:**

Our findings suggest that the studied SNPs on *ESR2* gene may not be associated with URSA.

## Introduction

Recurrent Spontaneous Abortion (RSA) is a multifactorial disorder and in most cases, a single cause for repeated abortions cannot be identified. RSA is defined as repeated occurrence of 3 or more miscarriages before 20^th^ week of gestation which affects about 0.5–1% of total pregnancies ^1^. Diverse factors, including uterine anomalies, chromosomal abnormalities, endocrine and immune defects, thrombophilia and infections could be associated with increased risk of RSA ^2^. In addition, at least half of the cases with RSA have no anomaly in any applied diagnostic test and are considered as cases with Unexplained RSA (URSA). With increase in the number of abortions, maternal factors that affect embryo-endometrial interactions may become more and more involved in pregnancy failures ^3^.

The role of hormones in reproductive events has been extensively investigated, and alterations in the Hypothalamus-Pituitary-Ovarian (HPO) axis factors are shown to negatively affect fertility and pregnancy. Recent studies revealed that genetic polymorphisms affecting the function of genes involved in regulating HPO, could be associated with RSA ^4^.

Estrogens are steroid hormones that affect reproductive system in both female and male. Estrogens are mainly produced in ovaries and influence female reproduction in several aspects, including progesterone induction, endometrial proliferation and maintenance, fetoplacental function and maturation, as well as utero-placental circulation ^5^.

Estrogen action is mediated by Estrogen Receptors (ERs). The ERs are ligand activated transcription factors and belong to the steroid/retinoid receptor gene super family that also includes receptors for androgens, progesterone, glucocorticoids, mineralocorticoids, as well as thyroid hormone, retinoic acid and vitamin D ^6^. Two nuclear receptors for estrogen have been identified in humans; Estrogen Receptor α(ERα) and Estrogen Receptor β(ERβ), coded by *ESR1* (located on chromosome 6q25.1) and *ESR2* (on chromosome14q22–24) genes, respectively, each consisting of 8 exons. Although these two receptors have high homologies, they are expressed in a preferable but sometimes over-lapping modes and display functional similarities as well as differences, sometimes even opposite actions ^7^.

The essential roles of ERβ in normal ovulation efficiency ^8^ and in regulation of follicular growth and oocyte development ^9^ are well documented, while ERα has a key role in fertilization ^8^. The genetic variants of *ESR1* and *ESR2* genes and their associations with ovulatory dysfunction especially those with unknown causes and pregnancy outcomes ^10,11^, infertility and endometriosis ^12–14^ have been investigated. The aim of this study was to examine the role of three Single Nucleotide Polymorphisms (SNPs) in the *ESR2* gene, including +1082G/A (a silent transition in exon 5),+1730G/A (3’untranslated region of the exon 8) and one intron (rs1256030 C/T in intron2) in URSA among Iranian population.

## Materials and Methods

### Subjects

In this case-control study, 240 women (mean age; 33.3±0.4 years, BMI; 26.6±0.3) were included as the case group, who suffered from at least three consecutive abortions before 20^th^ week of gestation, and were referred to Avicenna Fertility Center, Tehran, Iran, for treatment of RSA. Standard diagnostic procedures including a detailed history, chromosomal analyses of peripheral blood lymphocytes, ultrasonography and hysterosalpinogography to detect uterine anomalies, hormone profiles on day 2–3 of the menstrual cycles, measurement of Follicle Stimulating Hormone (FSH), Luteinizing Hormone (LH), and Estradiol (E2) as well as investigation of thrombophilia, infections and immune factors were performed for all patients. Patients with anatomical, chromosomal, infectious, endocrine, thrombophilia and autoimmune causes including anti-phospholipid syndrome were excluded from the study.

The control group consisted of 102 healthy ethnically matched women (mean age; 39.2±0.6 years, BMI; 27.5 ±0.4) with two or more successful pregnancies and live births and no history of complicated pregnancies, miscarriages, still births, small gestational age fetuses, pre-eclampsia, ectopic pregnancy or preterm delivery.

The study was approved by Avicenna Research Institute’s (ARI) local ethics committee for medical research, and written consents were obtained from participating subjects before entry into the study.

### Biochemical assays

Serum level of E2 was measured by Enzyme Immunoassay (EIA) (Axis Shield, Dundee, UK). LH and FSH were measured by Chemiluminescent Immune Assays (CLIA) (Diasorin; Saluggia, Italy).

### Single nucleotide polymorphism genotyping

Peripheral blood samples were obtained from all subjects and analyzed for ESR2 gene polymorphisms; +1082G/A, +1730G/Aandrs1256030C/T. Genomic DNA was extracted from anti-coagulated peripheral blood, using a standard salting out procedure ^15^. Genotyping of the *ESR2* polymorphisms was determined by Polymerase Chain Reaction-Restriction Fragment Length Polymorphism (PCR-RFLP), using specific primers as shown in table 1. PCR reaction mixtures contained 200 *ng* of DNA, 1xPCR Master Mix (Taq DNA Polymerase Master Mix Red, Ampliqon, Herlev, Denmark) and 10p moles of each specific primer. Annealing temperatures of 55.0*°C* for+1082G/A, 59.0*°C* for +1730G/A and 56.0*°C* for rs1256030C/T were used. The amplification cycles included 95*°C* for 5 *min*, followed by 35 cycles of 95*°C* for 30 *s*, 30 *s* for annealing and 72*°C* for 30 *s* and a final elongation time of 72*°C* for 5 *min*. PCR products were then digested by restriction endonucleases RsaI for +1082G/A, AluI for+1730G/A and rs1256030C/T (Fermentas, Vilnius, Lithuania) for 3 *hr* at 37*°C* (Table 1). Restriction endonuclease fragments were blended with gel red (Biotium, CA, USA) and then applied on to a 2–2.5% agarose gel and electrophoresed. Detection was made by visualization on an ultraviolet light transilluminator.

**Table 1.** Characteristics of studied SNPs on ESR2 gene

	**+1082 G/A**	**+1730 G/A**	**rs1256030 C/T**

**Region**	**Exon5**	**3′UTR, exon 8**	**Intron 2**
**PCR primers Upstream**	5′-TTGCGCAGCTTAACTTCAAA-3′	5′-GGTTTAGGGGTGGGGTAGACTG-3′	5′-CAATGCATATCCTGCCTGTG-3′
**Downstream**	5′-ACCTGTCCAGAACAAGATCT-3′	5′-GTGGAGGGAAGGATGGTACA-3′	5′-TCCCGGAAATCTGATACAGC-3′
**PCR product, *bp***	321 *bp*	442 *bp*	251 *bp*
**Digestion**	RsaI, 37*°C*	AluI, 37*°C*	AluI, 37*°C*
**Allele size, *bp***	G: 321^A^ A: 211+110^B^	G: 442^A^ A: 336+106^B^	C: 227+ 24^A^ T: 80+147+24^B^

A) RFLP product for normal allele;

B) RFLP product for mutant allele

### Statistical analysis

All statistical analyses were carried out with SPSS software package 16.0 (SPSS Inc., USA).The distributions of polymorphisms were compared between groups using Mann-Whitney U test. T-test was used to compare hormone levels between the groups. For comparisons between different genotypes and the levels of investigating hormones one way ANOVA was used. Correlations between different genotypes were determined with Spearman’s rank correlation test. Data was presented as mean±SEM and p-values <0.05 were regarded as significant.

## Results

Table 2 summarizes the hormone profiles of the study subjects. There were no significant differences between URSA and control groups in levels of E2 and FSH. Serum LH levels in the URSA group (although within normal range) were significantly higher than that in the controls (p<0.001).

**Table 2.** The hormone profiles of control and URSA women on day 2–3 of the menstrual cycles

**Characteristics**	**Controls**	**URSA**	**p-value^+^**
**Estradiol (*pg/ml*)**	44.5±6.0	37.4±3.6	NS
**FSH (*IU/ml*)**	7.0±0.4	7.5±0.3	NS
**LH (*IU/ml*)**	3.0±1.4	4.5±0.2	p<0.001

+ Determined by t-test. The data is presented as mean±SEM.

NS, not significant

The genotype frequencies of all subjects are shown in table 3. The frequency of +1082G/A polymorphism showed a weak trend to be significantly different in URSA compared to that in the control group (p=0.082). A homozygous +1082AA genotype was not found in any group. No significant differences in the genotype frequencies of +1730G/A andrs1256030C/T polymorphisms between the studied groups were observed (p= 0.519 and p=0.936, respectively).

**Table 3.** Genotype frequencies of ESR2 gene polymorphisms in URSA and control groups

**Polymorphism**	**URSA, no (%)**	**Control, no (%)**	**p-value ^†^**
**+1082G/A(rs1256049)**	n=237	n=102	0.082
**GG**	218(92%)	99(97.1%)	
**GA**	19(8%)	3(2.9%)	
**AA**	0.0%	0.0%	
**+1730 G/A(rs4986938)**	n=235	n=102	0.519
**GG**	104(44.2%)	48(47%)	
**GA**	108(46%)	32(31.4%)	
**AA**	23(9.8%)	22(21.6%)	
**rs1256030 C/T**	n=238	n=99	0.936
**CC**	56(23.5%)	27(27.3%)	
**CT**	110(46.2%)	38(38.4%)	
**TT**	72(30.3%)	34(34.3%)	

† Determined by Mann–Whitney U-test

Also, the subjects were further grouped into wild type, heterozygotic and homozygotic genotypes and the serum hormone levels within those groups were compared (Table 4). No statistically significant differences between the serum levels of measured hormones and the genotypes of the studied genetic polymorphisms were detected. However, a trend to lower serum FSH levels in women with the GA and AA genotypes compared with those with GG genotype of the *ESR2*+ 1730G/A was detected (p=0.063) (Table 4). In addition, in +1730G/A polymorphism, GA and AA genotypes also showed a trend to higher E2 compared to GG genotype (p=0.079) (Table 4).

**Table 4.** The hormone profiles of wild type, heterozygotic and homozygotic genotypes of ESR2 polymorphisms

	**Wild type**	**Heterozygous**	**Homozygous**	p-value^*^
**Estradiol (*pg/ml*)**				
+1082G/A	40.1±3.1	27.3±11.4	-	0.321
+1730 G/A	33.1±3.1	42.9±4.9	55.0±16.5	0.079
rs1256030 C/T	47.0±7.2	38.1±4.1	35.2±5.7	0.346
**FSH (*IU/ml*)**				
+1082G/A	7.3±0.2	8.4±0.8	-	0.301
+1730 G/A	7.8±0.3	7.3±0.4	6.0±0.5	0.063
rs1256030 C/T	7.3±0.4	7.7±0.4	6.8±0.3	0.296
**LH (*IU/ml*)**				
+1082G/A	4.1±0.2	5.5±0.8	-	0.099
+1730 G/A	4.1±0.4	4.3±0.2	3.7±0.6	0.722
rs1256030 C/T	3.9±0.4	4.2±0.3	4.2±0.4	0.790

Data presented as mean±SEM.

* Determined by one way ANOVA.

By using Spearsman’s rank correlation test, correlations between investigated polymorphisms were estimated. A negative correlation was found between polymorphisms; +1082G/A and +1730G/A (r=−0.137, p= 0.012). In addition, a positive correlation between+ 1730G/A and rs1256030C/T polymorphisms was detected (r=0.164, p=0.003). After subdividing case and control groups, the positive correlation between +1730 G/A and rs1256030C/T polymorphisms was only found in the control group (r=0.352, p<0.001) and the negative correlation between +1082G/A and +1730 G/A was only found in the URSA group (r=−0.155, p= 0.017).

## Discussion

RSA is recognized as a syndrome with a multifactorial etiology. At least, 50% of RSA cases were defined as RSA with unexplained origin. Investigating genes and biological mechanisms that can affect miscarriage may have beneficial effects on increasing live birth rates in cases suffering from URSA. Through investigating polymorphic genetic markers, a number of candidate genes for this syndrome have been identified. Genetic variation concerning thrombophilic and vascular genes is reported as a significant contributor to pregnancy complications ^2,16–18^. Polymorphisms in the estrogen receptor genes could affect different estrogen dependent pathways which may influence vascular tone and flow, leading to disruption of pregnancy maintenance. Role of *ESR* gene polymorphisms in increasing the risk of RSA has been investigated and most of the studies considered the involvement of *ESR1* gene polymorphism in this process ^19–21^. The role of *ESR2* gene polymorphisms in RSA was investigated in Brazil population, in 75 women with a history of RSA and 139 controls; in China population, in 196 women with RSA and 182 controls; and in Korean women, in 305 RSA women and 299 controls ^20,22,23^. No association between +1730G/A and +1082G/A polymorphisms with the syndrome was reported. Here, an attempt was made to search for a relation between three *ESR2* gene polymorphisms and URSA in Iranian women, a different ethnic group from above mentioned groups. But, no association between +1730G/A, +1082 G/A and rs1256030C/T polymorphisms and URSA could be shown. However, our study showed a trend to significant differences in +1082G/A polymorphism between URSA and control women.

Regarding the +1082G/A polymorphism, only two genotypes (GG and GA) were present in our study subjects which is in line with the study by Alessio *et al* who neither reported the presence of +1082AA in Caucasian controls nor in the RSA patients ^20^. Looking into genotypes and ethnicity, there are notable ethnic differences in the genotype distribution of +1082G/A polymorphism between Asians and Caucasians. Results from the present study showed that, similar to *ESR1* polymorphism study ^21^, the prevalence of Iranian genotype is more like Caucasians ^4,20^, which is different from Asians ^10,22^.

Considering the importance of studying +1082G/A and +1730G/A polymorphisms in *ESR2* gene, although such polymorphisms do not lead to amino acid changes in the ERβ protein, it is possible that these polymorphisms are in linkage disequilibrium with different regulatory sequence variations that may influence gene expression or function ^24^. Alternatively, some studies explained the functional influence of 3′-UTR-located SNPs on gene expression and local RNA structure and considered them as the cause of disease-related SNPs in non-protein-coding transcribed sequences ^25^. In addition, 3′-UTRs of a large number of protein-coding genes are shown to be objects for microRNAs. But, none of them happened on the 3′-UTR sequences of *ESR2* gene where +1730G/A is placed, although such microRNA may be present, but remains to be detected.

Furthermore, an intronic mutation may influence variable splicing and lead to a different final protein. In this regard, the intronicrs1256030C/T polymorphism has been associated with the urinary excretion levels of LH ^26^ and elevated risk of ovarian cancer ^27^. However, no association was found between this SNP and URSA.

Endocrinological abnormalities are present in about a quarter of women with unexplained recurrent miscarriage ^28^. In our study, although the URSA women had LH levels within laboratory normal serum range, a significant difference in the serum levels of LH was found between URSA and controls. Such findings confirm previous reports that hypersecretion of LH is associated with early pregnancy loss ^29,30^. However, genetic variants of the studied polymorphisms showed no significant association with the levels of LH. Lower amount of FSH might be harmful for ovulation induction. Here, a trend to higher E2 and lower FSH levels was found in women with the +1730 AA genotypes. In these women, the increased serum E2 level may suppress the pituitary in producing FSH. This finding may indicate the significance of screening URSA women for this SNP. This genotype has been suggested to be included in a screening panel for assessment of cardiovascular risk in menopausal women ^31^.

## Conclusion

In conclusion, our data suggest that *ESR2* gene in +1082G/A, +1730G/A and rs1256030C/T polymorphisms may not be involved with the risk of URSA, although further investigations regarding +1082G/A and +1730G/A genotypes are required to establish a role for URSA.
